# Absolute Pitch and Musical Expertise Modulate Neuro-Electric and Behavioral Responses in an Auditory Stroop Paradigm

**DOI:** 10.3389/fnins.2019.00932

**Published:** 2019-09-06

**Authors:** Vivek V. Sharma, Michael Thaut, Frank Russo, Claude Alain

**Affiliations:** ^1^Music and Health Research Collaboratory, University of Toronto, Toronto, ON, Canada; ^2^Rotman Research Institute, Baycrest Centre, Toronto, ON, Canada; ^3^Department of Psychology, Ryerson University, Toronto, ON, Canada; ^4^Department of Psychology, University of Toronto, Toronto, ON, Canada

**Keywords:** music, EEG, conflict resolution, event-related potentials, absolute pitch

## Abstract

Musicians have considerable experience naming pitch-classes with verbal (e.g., Doh, Ré, and Mi) and semiotic tags (e.g., musical notation). On the one end of the spectrum, musicians can identify the pitch of a piano tone or quality of a chord without a reference tone [i.e., absolute pitch (AP) or relative pitch], which suggests strong associations between the perceived pitch information and verbal labels. Here, we examined the strength of this association using auditory versions of the Stroop task while neuro-electric brain activity was measured using high-density electroencephalography. In separate blocks of trials, participants were presented with congruent or incongruent auditory words from English language (standard auditory Stroop), Romanic solemnization, or German key lexicons (the latter two versions require some knowledge of music notation). We hypothesized that musically trained groups would show greater Stroop interference effects when presented with incongruent musical notations than non-musicians. Analyses of behavioral data revealed small or even non-existent congruency effects in musicians for solfège and keycodes versions of the Stroop task. This finding was unexpected and appears inconsistent with the hypothesis that musical training and AP are associated with high strength response level associations between a perceived pitch and verbal label. The analyses of event-related potentials revealed three temporally distinct modulations associated with conflict processing. All three modulations were larger in the auditory word Stroop than in the other two versions of the Stroop task. Only AP musicians showed significant congruity effects around 450 and 750 ms post-stimulus when stimuli were presented as Germanic keycodes (i.e., C or G). This finding suggests that AP possessors may process alpha-numeric encodings as word forms with a semantic value, unlike their RP possessing counterparts and non-musically trained individuals. However, the strength of musical conditional associations may not exceed that of standard language in speech.

## Introduction

Musicians have considerable experience naming pitch-classes with verbal and semiotic tags. A small subset of musicians possesses linguistic tags for decoding pitch-classes into verbal codes or symbols without having to resort to an external reference pitch. This ability to name pitches without a referent is called absolute pitch (AP). The phenomenon of AP is of particular interest because it links measurable sensory inputs to neural representations (Zattore, 2003). AP also demonstrates highly stable category judgments using verbal codes that the general population does not seem to have for isolated sound objects (Levitin and Rogers, 2005). Most musicians do not have AP and instead require an external pitch-class to be presented and maintained in working memory as a reference to compare a subsequent pitch-class in order to label it. This more common ability is referred to as relative pitch (RP), which is the ability to label the intervals between pitches. Thus, while AP musicians can name pitch categories without any external referent, RP musicians can name pitch categories once a reference pitch is provided.

Absolute pitch is thought to be “automatic” whereas almost no RP users report automatic responses when identifying a pitch-class on the basis of knowledge of a preceding one, possibly relying on focused attention and working memory (Zatorre et al., 1998). AP possessors can accurately name a pitch-class within less than a second of hearing it (Miyazaki, 1990). Evidence also shows that AP is likely acquired during a critical period of 5–7 years of age (Russo et al., 2003; Miyazaki and Ogawa, 2006). There is also a negative correlation between mean response time (RT) and accuracy across participants in AP groups (Takeuchi and Hulse, 1993). Musicians with RP are faster at naming the intervals between two pitch-class categories, most likely because AP possessors are thought to name individual elements first then extrapolate distance, adding processing stages to their interval judgments (Miyazaki, 1992). In contrast, RP can be developed by most individuals at any age and can become quite rapid and accurate with training and rehearsal (Miyazaki, 1989). Musicians with RP cannot associate abstract entities such as verbal codes to individual pitches consistently over a long-term but only to intervals or chord structures. AP possessors are quicker and most accurate when responding to piano-tones, the instrument of acquisition for most musicians with AP – some can only label piano-tones (Miyazaki, 1989). However, AP possessors have more difficulties naming individual pitch when presented with “black-key” pitch classes or simple tones (Takeuchi and Hulse, 1993). Interestingly, musicians with AP may also be susceptible to imperceptibility of “key signature” – the macroscopic organizational schema for all pitches in a piece of music, where a single pitch is perceived as the 1st element of the musical scale sequence (Terhardt and Seewann, 1983). Baharloo et al. (1998) delineated four categories of AP, where certain categories had high accuracy and low RTs to sinetone tests, while other categories did not, which informed the level of automaticity expected in individual AP possessors. Currently, it is thought that non-AP requires working memory to keep reference tones and schema of tonal intervals in echoic memory and consciousness while AP rely more heavily on categorization networks during pitch naming (Schulze et al., 2009). Thus, RP uses working memory and conscious control while AP seems to be automatic and working memory free. In this sense, much of RP ability is schematic while AP may employ more stimulus driven responses.

In the present study, we used auditory versions of the Stroop task to explore how pitch may be internally represented in musicians with RP and AP. In the original version of the Stroop task, participants are presented with a colored word, which could either be congruent (e.g., the word RED in red ink), incongruent (e.g., the word RED in blue ink), or neutral (e.g., word printed in black or a series of the letter “X” printed in colors). Participants are slower and make more errors for incongruent than congruent or neutral stimuli (MacLeod, 1991). The Stroop task has been used to study conflict resolution in children (Prevor and Diamond, 2005) and older adults (West and Alain, 2000) as well as in neurological (Rafal and Henik, 1994) and psychiatric (McNeely et al., 2003) populations. In the auditory versions of the Stroop task, participants are presented with spoken words that could either be congruent (e.g., the word Low in low pitch) or incongruent (e.g., the word Low in high pitch) (Hamers and Lambert, 1972). As with the visual Stroop task, participants are slower and make more errors for incongruent than congruent stimuli (Itoh et al., 2005; Donohue et al., 2012). If musicians process musical annotations similarly to how the general population process words, then we should observe interference effects when they are presented with incongruent musical annotations. However, it is unclear how musicians might process incongruence between semantic meaning and linguistic information during category recognition of musical pitch-classes and notations. Musical experience may allow for behavioral flexibility in an auditory Stroop paradigm involving pitch information. For example, musicians with AP may attenuate or bypass conflicting linguistic information during pitch perception, biasing their attention toward their own internal words. Thus, developing associative bonds between language and pitch may generate reflexive, rapid, and accurate encoding of sensory features irrespective of conflicting verbal codes. That is, audible musical percepts with strongly associated codewords may be less susceptible to interference than associated verbal codes outside of the domain of music. This type of transfer effect has been discussed in the context of musical expertise by some researchers, who suggest that scalp recording of event-related potentials (ERPs) may be effective to disentangle neural time-course events to study these effects (Besson et al., 2011).

Earlier ERP studies of the Stroop effect have identified several neural correlates thought to play an important role in information conflict resolution. For example, Duncan-Johnson and Kopell (1981) found that the P300 response at midline scalp locations (i.e., Fz, Cz, and Pz) was not significantly modulated by semantic incongruence or neutrality of color and word and yet the classic Stroop stimuli did indeed elicit a typical Stroop effect, which suggested that based on their resulting RTs, the neural signature of Stroop interference should more likely rely on activity later than the P300 response. This was also supported by other investigators (Ilan and Polich, 1999; Atkinson et al., 2003). Rebai et al. (1997) demonstrated that a late negative N400 wave is evoked when participants are asked to internally verbalize responses of incongruent color-word conditions from recordings of the Fz, Cz, and Oz plus left and right parietal electrodes. West and Alain (1999) noted four spatially and temporally distinct modulations during the visual Stroop task, which included a phasic polar positivity that peaked at about 500 ms after stimulus onset over lateral fronto-polar scalp areas, a fronto-central slow wave that began at about 500 ms after the stimuli and persisted over the remainder of trial, a left-parietal modulation peaking at 522 ms and a left temporo-parietal positivity beginning at about 600 ms after stimulus onset. Ergen et al. (2014) showed that the N450 wave was larger for incongruent stimuli in comparison to congruent stimuli and maximal over frontal electrodes. These investigators also found a slow late potential at around 600 ms after stimuli onset over the parietal electrodes of their extended 10/20 system (Ergen et al., 2014).

In the present study, non-musicians (NM) and musicians with AP or RP completed three different versions of an auditory Stroop task while we measured neuro-electric brain activity using high-density electroencephalography. In the auditory word Stroop task, participants were presented with sung English words (i.e., low or high) in one of two pitches corresponding semantically to the words. They were told to ignore the word and press a button indicating the pitch of the stimulus. In the solfège version of the auditory Stroop task, participants were instructed to do the same as the word Stroop task. However, they were presented with the English solemnizations corresponding to the two pitches (i.e., Doh or Soh). Finally, in the keycodes task, participants were presented with phonemic equivalents of key notation that corresponded to the two pitches (i.e., “C” and “G”) and instructed to do the same as the other two tasks. For the word Stroop task, we anticipated that musicians would be faster and more accurate than NMs, but that the Stroop interference effects should be similar in all three groups. For the solfège and keycodes versions of the Stroop task, we predicted greater Stroop interference in musicians with RP and AP due to an over-learned semantic response and prolonged exposure to congruencies from pitch naming training. Prior research using auditory word Stroop (Itoh et al., 2005; Donohue et al., 2012) found analogous neuro-electric modulations to those found in the visual domain in terms of increased negativity for incongruent compared to congruent conditions over midline central scalp area between 400 and 600 ms after stimulus onset (West and Alain, 1999). We anticipate a similar modulation in NMs but a possible attenuation of such responses in musically trained individuals for pitch naming-based Stroop effects that use non-musical standard English words. Finally, we hypothesize that musicians with RP and AP would show enhanced negativity for incongruent trials in the solfège and keycodes tasks because of strong internal pitch representations and prolonged exposure to pitch naming with these lexicons. Understanding the strength of pitch information to verbal code mappings may demonstrate that creating abstract representations of perceptual features may allow confirmation of what is heard with greater certainty. This may help explain how words tag onto object features in order for categories to emerge from the environment.

## Materials and Methods

### Participants

Sixty participants were recruited for this study. One NM was excluded, one musician with AP and one musician with RP were excluded because of excessive eye movements during EEG recording. The final sample comprised 19 NM (mean age = 24.9 years, range = 20–39, *SD* = 4.3, five males) with <4 years exposure to any music training. It also included 19 musicians who possess AP (mean age = 25.1 years, range = 19–41, *SD* = 5.9, five males) and 19 who possess RP (mean age = 26.4 years, range = 21–39, *SD* = 4.0, 5 males) all of whom reported >7 years of formal musical training. There were no significant differences between the groups for age and years of education.

### Screening Test

#### Stimuli and Apparatus

Stimuli consisted of 24 pure tones (44.1 kHz sampling rate). They were generated using the digital audio editing software, ProTools LE (Version 7.3.1, Daly City, CA, United States). All stimuli were 1000-ms in duration, including 5-ms onset and 30-ms linear offset ramps. Pitch-classes were equally spaced, ranging from C3 to B5 inclusively and tuned to standard A4 = 440 Hz piano tunings in equal temperament. The stimuli were presented binaurally in an order that was individually randomized for each participant.

A computer interface presented the stimuli and collected response data from the participants using customized software in MATLAB by Mathworks (Cambridge, MA, United States, 2015b). Participants were tested in a quiet room in front of the computer interface, which was equipped with a mouse that allowed the participants to classify the tones using 12 circular response regions. Each of these regions displayed a complete set of chromatic pitch-class labels for AP and diatonic for RP, including enharmonic equivalents. The response-regions were placed in a circular shape, forming a clock of pitch-class labels. A small circle was displayed around the center of this wheel for the participants to click within before hearing a tone. Category response regions were positioned such that each response was equidistant from the pointing cursor at the onset of every tone.

For RP musicians, the stimuli and apparatus were the same, except the pitch C4 was always presented preceding the pitch meant to be named. Because RP is considered to be more effortful and less acute than AP, black-key pitches were not included nor were their pitch-class labels presented on screen, which decreased the response set to ease working memory load. The RP test consisted of naming 12 stimuli ranging based on their distance from C4.

#### Screening Procedure

We used the screening procedure developed by Bermudez and Zatorre (2009). The screen tests employed the same computer and digital audio system as the experiment. For the purposes of this study, the criterion for AP was defined as the ability to score ≥85% or an absolute mean deviation of ≤1 under these screening test conditions. RP was defined as the ability to score ≥80% and participants with mean RT that were three standard deviations from the group mean were eliminated. A set of structured questionnaires and follow-up interviews were then administered after testing to index musical experience and health-related data.

### Experimental Test

#### Stimuli

The auditory stimuli were seven sung monosyllabic phonemes: /low/, /high/, /do/, /so/, /see/, /jee/, and /baw/. A professional vocalist sung the phonemes, which were recorded at a sample rate of 44,100 Hz using a Shure SM58 microphone and ProTools LE (Version 7.3.1, Daly City, CA, United States). The fundamental frequency of each tone was transformed digitally to be tuned to 261.63 Hz for the low pitch and 392 Hz for the high pitch using Adobe Audition 1.5 (San Jose, 2004). Sinusoid tones were generated with the same frequencies as the sung speech tones (i.e., C4 = 261.63 Hz and G4 = 392 Hz). The /baw/ syllable was recorded in both pitches and used as a neutral phoneme for all lexicons. Average root mean square power of the tones was then normalized (*M* = −16.13 dBs, *SD* = 1.72 dBs). The stimuli were edited to have a 500-ms duration including 5-ms rise/fall time. All phonemes were standardized to the Carnegie Melon University Pronouncing Dictionary. Stimuli were presented binaurally at an intensity of 75 decibels sound pressure level (SPL) through insert earphones model ER-3A by Etymotic Research (Elk Grove Village, 1985).

#### Procedure

Participants sat in a comfortable chair in an acoustically and electrically shielded room. The stimuli were presented using the computer software Presentation 16.5 by Neurobehavioral Systems Inc. Trials consisted of 500-ms fixation cross, followed by 500-ms auditory stimulation. The fixation cross was maintained on screen throughout the stimulus, and for 500-ms after a response was made, which initiated the next trial. The inter-trial interval was 1000-ms. The fixation cross was a white cross with a 58 point font size and located in the center of a black screen. Each block of trials comprised 100 congruent, 100 incongruent, and 67 neutral trials, which were presented in completely random order within each block. Participants completed three blocks of trials with breaks offered between blocks. They responded using a computer keyboard and were instructed to press the left arrow key for “Low” pitch and the right arrow key for “High” pitch, regardless of sung word. The time between the onset of a tone and a key press was recorded as the RT for that trial. RTs that were greater than 1000-ms or less than 200-ms were excluded from further analyses.

### EEG Acquisition and Preprocessing

Electroencephalogram (EEG) was recorded from 66 scalp electrodes using a BioSemi Active Two acquisition system (BioSemi V.O.F., Amsterdam, Netherlands). The electrode montage was set according to the BioSemi electrode cap based on the 10/20 system and included a common mode sense active electrode and driven right leg passive electrode serving as ground. Ten additional electrodes were placed below the hair line (both mastoid, both pre-auricular points, outer canthus of each eye, inferior orbit of each eye, two facial electrodes) to monitor eye movements and to cover the whole scalp evenly. The latter is important because we used an average reference (i.e., the average of all scalp EEG channels as the reference for each EEG channel) for ERP analyses. Neuro-electric activity was digitized continuously at a rate of 512 Hz with a bandpass of DC-100 Hz, and stored for offline analysis. All off-line analyses were performed using Brain Electrical Source Analysis software (BESA, version 6.1; MEGIS GmbH, Gräfelfing, Germany).

The continuous EEG data were first digitally filtered with 0.3 Hz high-pass (forward, 6 dB/octave) and 40 Hz low-pass filters (zero phase, 24 dB/octave). For each participant, a set of ocular movements was identified from the continuous EEG recording and then used to generate spatial components that best account for eye movements. The spatial topographies were then subtracted from the continuous EEG to correct for lateral and vertical eye movements as well as for eye-blinks. The analysis epoch consisted of 200 ms of pre-stimulus activity and 1000 ms of post-stimulus activity time-locked to the stimulus onset. After correcting for eye movements, data for each participant were then scanned for artifacts; epochs including deflections exceeding 120 μV were marked and excluded from the analysis. The remaining epochs were averaged according to electrode position, stimulus type (i.e., neutral, congruent, incongruent), experimental condition (i.e., auditory word Stroop, Solfège, and Keycodes), and response accuracy (correct vs. incorrect response). Each average was baseline-corrected with respect to the pre-stimulus interval (i.e., mean amplitude over the 200 ms prior to stimulus onset).

The ERP analyses involved only correct trials and focused on mean amplitude for three pre-defined time windows and electrode clusters motivated by prior auditory (i.e., Itoh et al., 2005; Donohue et al., 2012) and visual (West and Alain, 1999) Stroop studies. The first window (150–250 ms) comprised of C1, Cz, C2, FC1, FCz, and FC2 electrodes over the fronto-central scalp area to measure the P200 response. The N450 (325–525 ms) was quantified using the same electrodes. A late positive component (LPC) modulation (600–900 ms) was quantified over the P1, P3, Pz, P2, P4, PO3, POz, and PO4 electrode sites.

## Results

In a preliminary analysis we examined whether RTs and error rates differed for the two neutral stimuli (i.e., phonemes and sinetones). For RTs, the ANOVA with Neutral Type and Task as within subject factors and Group as between-subject factors did not reveal significant differences in RT as a function of Neutral Type [*F*(1,54) = 3.16, *p* = 0.081], nor did this factor interact with Musicianship [*F*(2,54) = 2.23, *p* = 0.118] or Task (*F* < 1). The three way interaction between Neutral Type, Task, and Musicianship was not significant [*F*(4,108) = 1.04, *p* = 0.391]. For error rates, the ANOVA with Neutral Type and Task as within-subject factors and Group as between-subject factors did not reveal significant differences in error as a function of Neutral Type [*F*(1,54) = 2.96, *p* = 0.091], nor did this factor interact with Musicianship [*F*(2,54) = 1.18, *p* = 0.313] or Task (*F* < 1). The three way interaction between Neutral Type, Task, and Musicianship was not significant [*F*(4,108) = 1.18, *p* = 0.322]. Thus, the RTs and error rates for neutral phonemes and sinetones were averaged together into a single neutral condition.

### Musicianship and Congruency Effect on Response Time

**FIGURE 1:**
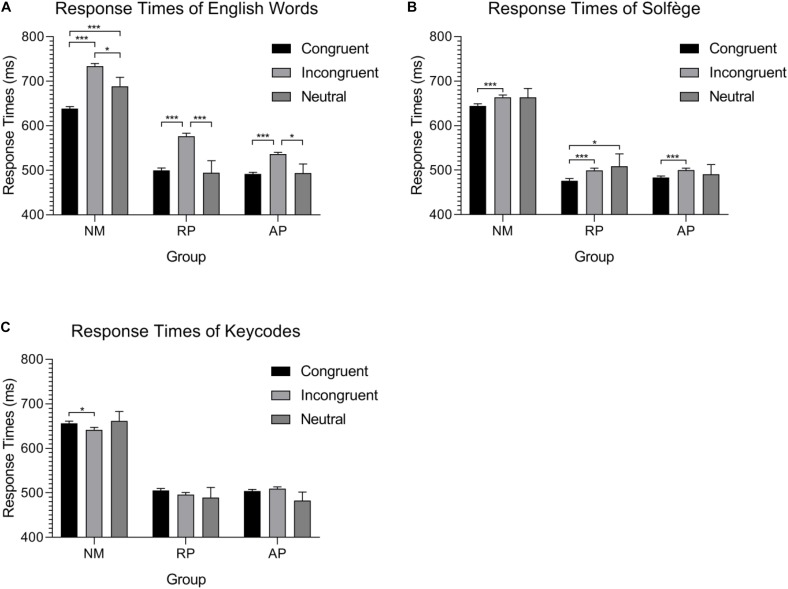
Group mean response time during the three auditory Stroop tasks. **(A)** Musicians and non-musicians process neutral stimuli differently during the English words task. **(B)** Significant differences between congruent and incongruent stimuli in the solfège task show a possible facilitation effect of the lexicon but still demonstrate interference. **(C)** A possible reverse behavioral Stroop effect for alpha-numeric codes is found for isolated alpha-numeric codes from color-words in NMs, though the effect is marginal. No other differences are found for this task. ^∗^*p* < 0.05, ^∗∗^*p* < 0.01, and ^∗∗∗^*p* < 0.001.

The group mean RTs for each task and condition are shown in Figure 1. A mixed design ANOVA on RTs with Musicianship (NM, RP, and AP) as a between-subject factor and Task (Words, Solfège, and Keycodes) and Congruence (Congruent, Incongruent, and Neutral) as within-subject factors and *post hoc* comparisons used the Bonferroni procedure. The ANOVA yielded a three-way interaction between Musicianship, Task, and Condition [*F*(8,216) = 2.81, *p* < 0.01, ${\text{η}}_{\text{p}}^{2}$ = 0.094]. To better understand this interaction, we examined the effect of musicianship and condition in each task separately.

For the Words task, we anticipated that musicians would be faster than non-musicians given the mounting evidence that musical training enhanced executive function and cognitive control (Moussard et al., 2016; Alain et al., 2019). The ANOVA on RTs revealed a significant group × stimulus type interaction [*F*(4,108) = 5.56, *p* < 0.001, ${\text{η}}_{\text{p}}^{2}$ = 0.171], implying differentials of interference effects between the AP, RP, and NM groups. Nonetheless, it should be noted that the main effect of musicianship was significant [*F*(2,54) = 20.97, *p*<0.001, ${\text{η}}_{\text{p}}^{2}$ = 0.437]. *Post hoc* comparisons show that there was no difference between AP and RP (*p* = 1) though both groups were significantly faster than NMs (*p* < 0.001 for both differences) due to an overall RT advantage for this task, despite congruency effects. As for the interaction between congruence and musicianship, NMs were significantly slower for incongruent trials, intermediate for neutral, and fastest for congruent trials (all pairwise comparisons were significant, *p* < 0.01 in all cases). For musicians with AP, the congruent condition was significantly faster than incongruent (*p* < 0.001) but not neutral (*p* = 1), and the RTs to neutral words were significantly faster than those generated for incongruent stimuli (*p* < 0.01). Finally, musicians with RP showed slower RTs to incongruent than both congruent (*p* < 0.001) and neutral (*p* < 0.001) stimuli, but no difference was seen between congruent and neutral.

For the Solfège task, we expected NMs to show little interference since they do not associate verbal codes with pitch representation using this lexicon, while musicians should show interference, particularly AP who have learned to associate pitch representations with labels automatically. Surprisingly, the interaction between musicianship and congruency was not significant. This suggests comparable interference effect in AP, RP, and NM groups. Nonetheless, the main effect of musicianship was significant [*F*(2,54) = 21.05, *p* < 0.001, ${\text{η}}_{\text{p}}^{2}$ = 0.438], with AP and RP musicians not being significantly different in RT and both musicians groups being faster than NMs (*p* < 0.001 for both differences). There was also a main effect of congruence [*F*(2,54) = 6.68, *p* < 0.01, ${\text{η}}_{\text{p}}^{2}$ = 0.110]. Pairwise comparison shows that the congruent condition was significantly faster than both incongruent (*p* < 0.001) and neutral (*p* = 0.029), while no difference was found between incongruent and neutral.

For the Keycodes task, we expected NMs to also show little to no interference unlike musicians. The interaction between congruence and musicianship was significant [*F*(4,108) = 2.97, *p* = 0.023, ${\text{η}}_{\text{p}}^{2}$ = 0.099]. Again, the main effect of musicianship was significant [*F*(2,54) = 21.04, *p* = 0.001, ${\text{η}}_{\text{p}}^{2}$ = 0.438] in a similar manner as the other two tasks. The main effect of congruence was not significant. Surprisingly, the NM group was significantly faster for the incongruent condition when compared to the congruent condition (*p* = 0.012).

### Musicianship and Congruency Effect on Error Rates

**FIGURE 2:**
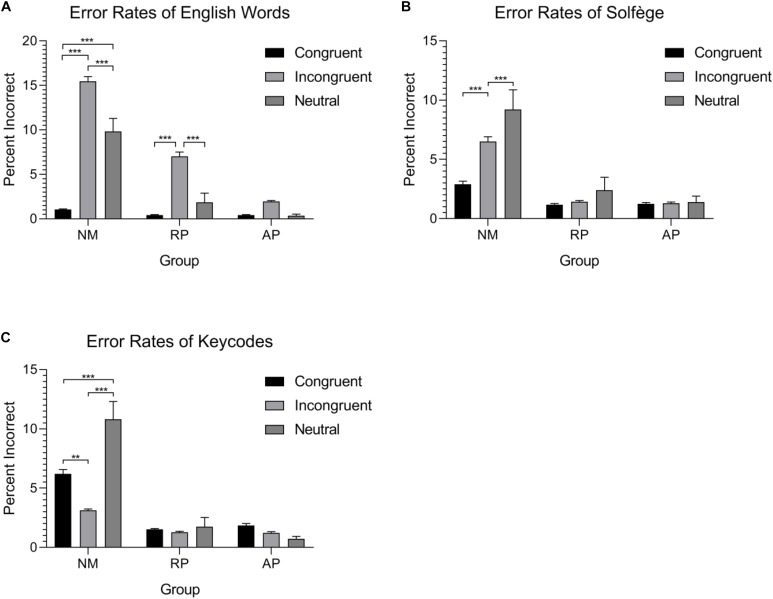
Error-rates for the three tasks and three groups. Both musician groups have significantly lower errors overall in comparison to the non-musician group. **(A)** AP possessors show very little errors for standard word form Stroop task. **(B)** Musicians show no differences in errors for the solfège lexicon. **(C)** Only non-musicians show considerable error rate differences within the conditions and a reverse Stroop effect between congruent and incongruent stimuli. ^∗∗^*p* < 0.01 and ^∗∗∗^*p* < 0.001.

The group mean error rates for each task and condition are shown in Figure 2. A mixed design ANOVA with the same factors as the RTs was performed for error rates. Similar to RTs, the three-way interaction between Musicianship, Task, and Congruency was significant [*F*(8,216) = 9.16, *p* < 0.001, ${\text{η}}_{\text{p}}^{2}$ = 0.253]. To better understand this interaction, we examined the effect of musicianship and condition in each task separately.

For English words, the interaction between musicianship and condition was significant [*F*(4,108) = 11.31, *p* < 0.001, ${\text{η}}_{\text{p}}^{2}$ = 0.295]. NMs were more accurate for congruent than incongruent or neutral stimuli (*p* < 0.001, in both cases). They were also more accurate for neutral than incongruent stimuli (*p* < 0.001). Musicians with RP were more accurate for congruent than incongruent stimuli (*p* < 0.01). They were also more accurate for neutral than incongruent stimuli (*p* < 0.01). There was no difference between the neutral and congruent words condition. Musicians with AP did not show significant differences in accuracy for congruent, incongruent, and neutral stimuli for this task.

For the Solfège task, there was a significant interaction between musicianship and condition [*F*(4,108) = 6.36, *p* < 0.001, ${\text{η}}_{\text{p}}^{2}$ = 0.191]. NMs were more accurate for congruent than incongruent or neutral stimuli (*p* < 0.001 in both cases). They were also more accurate for neutral than incongruent stimuli (*p* = 0.024). Musicians with RP and AP showed similar error rates in this task for congruent, incongruent, and neutral stimuli with no significant differences.

For the keycodes task, there was a significant musicianship by condition interaction [*F*(4,108) = 12.89, *p* < 0.001, ${\text{η}}_{\text{p}}^{2}$ = 0.323]. NMs were more accurate for incongruent than congruent (*p* < 0.01). They were also more accurate for incongruent and congruent than neutral stimuli (*p* < 0.001 in both cases). Musicians with RP and AP showed comparable error rates in the keycodes task with no significant accuracy differences between congruent, incongruent, and neutral stimuli.

### Effects of Musicianship on Neural Correlates of Stroop Effect

**FIGURE 3:**
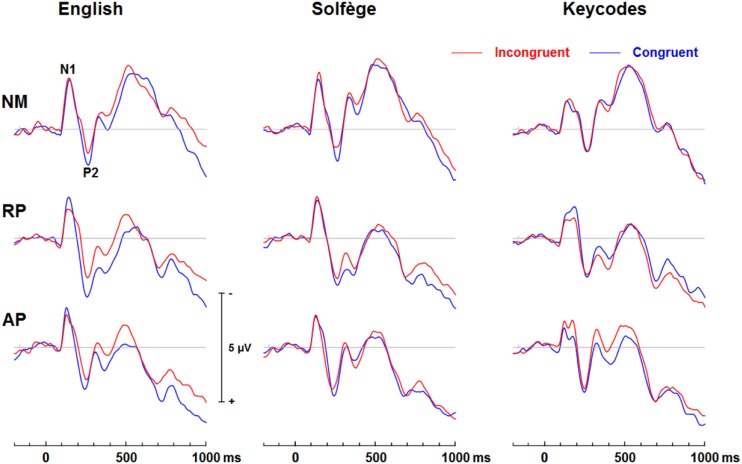
Event-related potential waveforms of congruent and incongruent conditions for each task at the FCz site. A clear N1 and P2 neural electric modulation is visible, along with an expected N450. English words show a congruency effect for all groups for the N450 but only AP shows this effect for keycodes.

**FIGURE 4:**
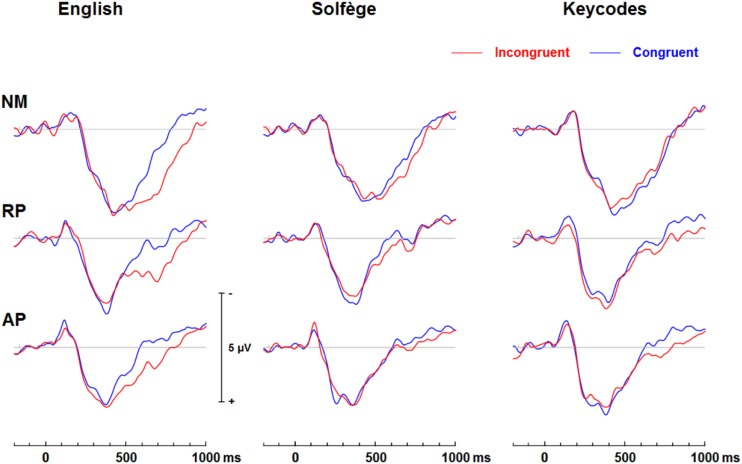
Event-related potential waveforms at the midline parietal (Pz) electrode site showing a posterior late positive complex (LPC) wave that significantly differs in amplitude at late stages of processing for congruent and incongruent word form conditions, which may reflect a post-trial motor response awareness. English words elicit significant congruence effects but only AP shows significant LPC differences for keycodes.

**FIGURE 5:**
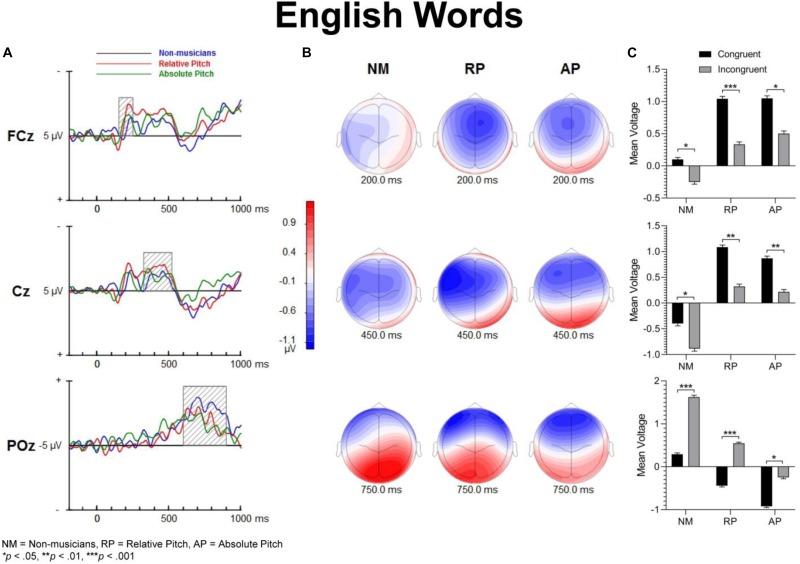
Event-related potential difference waveforms of incongruent minus congruent conditions and topographic maps. **(A)** Difference waves forms and shaded areas displaying temporal windows of interest. **(B)** Topographic maps show fronto-central negativities at 200 and 450 ms along with a posterior positivity at 750 ms post-stimulation. **(C)** Bar graphs showing group mean event-related potential amplitude for the P200 (top panel), N450 (middle panel) and LPC (bottom panel).

**FIGURE 6:**
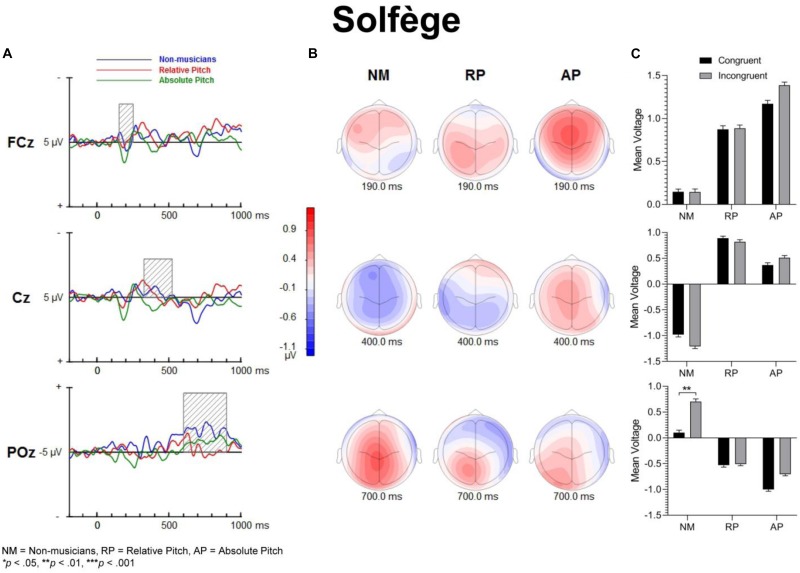
Event-related potential difference waveforms of incongruent minus congruent conditions and topographic maps. The solfège task does not necessarily evoke a strong inhibitory response, though the non-musicians have a post-trial positivity in their LPC potentials. **(A)** Difference waveforms and shaded areas displaying temporal windows of interest. **(B)** Topographic maps show difference dipoles at 190 and 400 ms along with a posterior positivity at 700 ms post-stimulation, which occur within the temporal windows of interest. **(C)** Bar graphs showing group mean event-related potential amplitude for the P200 (top panel), N450 (middle panel) and LPC (bottom panel).

**FIGURE 7:**
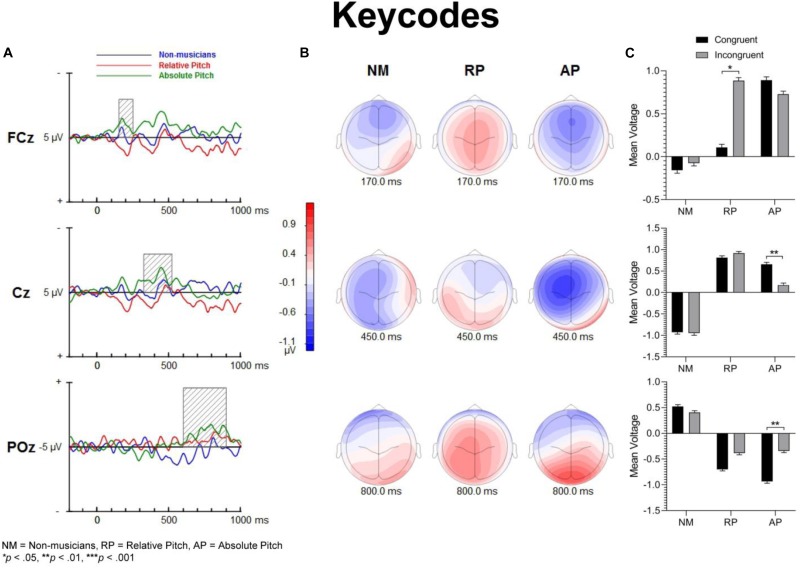
Event-related potential difference waveforms of incongruent minus congruent conditions and topographic maps. Both AP and NM groups show a fronto-central negativity for keycodes task, yet only AP have familiarity and also a significant differences of N450 and posterior positivity for this task. **(A)** Difference waves and shaded areas displaying temporal windows of interest. **(B)** Topographic maps show fronto-central negativities at 170 and 450 ms along with a posterior positivity at 750 ms post-stimulation. **(C)** Bar graphs showing group mean event-related potential amplitude for the P200 (top panel), N450 (middle panel) and LPC (bottom panel).

Figure 3 shows group mean ERPs elicited as a function of task and congruency. In all three groups, stimuli generated an N1 and P2 deflection at fronto-central sites, which inverted in polarity at inferior parietal sites and mastoids, consistent with generators in superior temporal gyrus. These sensory evoked responses were followed by a broad negativity that peaked at about 525 ms after sound onset at central scalp sites. This negativity was followed by a positive wave at parietal sites (Figure 4). The analyses of ERP amplitude focus on congruent and incongruent trials. The ERPs generated by the neutral trials were excluded because the neutral stimuli had distinctive acoustic features making it difficult to dissociate congruency effects from acoustically driven factors. Three different spatio-temporal clusters were examined: Central P200, Central N450, and Parietal LPC (Figures 5–7).

#### Central P200

Figures 5–7 show the group mean amplitude as a function of task and congruency. The three-way interaction between musicianship, task, and congruency was significant [*F*(4,108) = 3.00, *p* = 0.045, ${\text{η}}_{\text{p}}^{2}$ = 0.085]. To further elucidate this interaction, the tasks were analyzed separately in a similar manner as the behavioral results. The main effect of musicianship was not significant.

For English words, the interaction between musicianship and group was not significant. However, there was a main effect of congruency [*F*(1,54) = 42.93, *p* < 0.001, ${\text{η}}_{\text{p}}^{2}$ = 0.443], where the congruent condition was significantly more positive than the incongruent condition (*p* = 0.017 for NM, *p* < 0.001 for RP and AP). For the solfège and keycodes tasks, there were no significant main effects or interactions between group and congruence.

#### Central N450

Figures 5–7 display the differences in amplitudes for the N450 modulation. The three-way interaction between group, task, and congruency trended toward significance [*F*(4,108) = 2.12, *p* = 0.084], whereas the two-way interaction between task and congruency was significant [*F*(1,54) = 8.59, *p* < 0.001, ${\text{η}}_{\text{p}}^{2}$ = 0.141]. To further elucidate the three-way interaction, the tasks were analyzed separately. The ANOVA also yielded a main effect of congruency [*F*(1,54) = 20.29, *p* < 0.001, ${\text{η}}_{\text{p}}^{2}$ = 0.273], with greater negativity in ERPs for incongruent than congruent stimuli (*p* < 0.001) across the tasks. A significant main effect of musicianship was also found [*F*(2,54) = 5.18, *p* < 0.01, ${\text{η}}_{\text{p}}^{2}$ = 0.161]. Pairwise comparisons showed larger ERP amplitude in NMs compared to AP (*p* = 0.048) and RP (*p* = 0.010) across the tasks and congruencies.

For the English word Stroop task, the group by congruency interaction was not significant [*F*(2,54) = 0.371, *p* = 0.692, ${\text{η}}_{\text{p}}^{2}$ = 0.014]. In all three groups, the incongruent stimuli generated significantly greater negative ERP amplitude than the congruent stimuli [*F*(1,54) = 22.77, *p* < 0.001, ${\text{η}}_{\text{p}}^{2}$ = 0.297]. The main effect of musicianship trended toward significance [*F*(2,54) = 3.11, *p* = 0.053, ${\text{η}}_{\text{p}}^{2}$ = 0.103].

For solfège, the group by congruency interaction was not significant [*F*(2,54) = 1.60, *p* = 0.212, ${\text{η}}_{\text{p}}^{2}$ = 0.056]. However, there was a main effect of musicianship [*F*(2,54) = 6.85, *p* < 0.01, ${\text{η}}_{\text{p}}^{2}$ = 0.202]. Pairwise comparisons revealed larger ERP amplitude in NMs than musicians with RP (*p* < 0.01) or AP (*p* = 0.021). The ERP amplitude was comparable for the RP and AP groups and not significantly different.

In the keycodes task, there was a significant interaction between musicianship and congruency [*F*(2,54) = 3.67, *p* = 0.032, ${\text{η}}_{\text{p}}^{2}$ = 0.120]. Musicians with AP showed a significant congruency effect (i.e., N450, *p* < 0.01), while no differences in ERP amplitude were found between congruent and incongruent stimuli in NM and musicians with RP.

#### Parietal LPC (600–900)

Figures 5–7 show the differences in ERP waveform amplitudes for the parietal LPC. The three-way interaction between musicianship, task, and congruency was significant [*F*(4,108) = 2.67, *p* = 0.036, ${\text{η}}_{\text{p}}^{2}$ = 0.090]. Once again, to further elucidate this interaction, the tasks were analyzed separately. There was also a main effect of task [*F*(2,108) = 2.67, *p* = 0.036, ${\text{η}}_{\text{p}}^{2}$ = 0.090] and a main effect of musicianship [*F*(2,54) = 6.15, *p* < 0.01, ${\text{η}}_{\text{p}}^{2}$ = 0.186].

For the English words, the group by congruency interaction was not significant [*F*(2,54) = 1.64, *p* = 0.204, ${\text{η}}_{\text{p}}^{2}$ = 0.057]. Overall, the ERP amplitude was smaller for congruent than incongruent stimuli [*F*(1,54) = 45.53, *p* < 0.001, ${\text{η}}_{\text{p}}^{2}$ = 0.457]. The main effect of musicianship was significant [*F*(2,54) = 7.36, *p* < 0.01, ${\text{η}}_{\text{p}}^{2}$ = 0.214]. Pairwise comparisons reveal that the musicians with AP generated more negative amplitude than NM group (*p* < 0.01). There was no difference in ERP amplitude between the NM and RP group (*p* = 0.074).

In the solfège task, the group by congruency interaction was not significant [*F*(2,54) = 1.86, *p* = 0.166, ${\text{η}}_{\text{p}}^{2}$ = 0.064]. The ERP was significantly more positive in amplitude for congruent than incongruent stimuli [*F*(1,54) = 6.21, *p* = 0.016, ${\text{η}}_{\text{p}}^{2}$ = 0.103]. There was a main effect of musicianship [*F*(2,54) = 4.66, *p* = 0.014, ${\text{η}}_{\text{p}}^{2}$ = 0.147]. Pairwise comparisons showed greater positive ERP amplitude in NMs than musicians with AP (*p* = 0.013) but not RP (*p* = 0.086). There was no difference in LPC amplitude for the solfège task between AP and RP groups.

For keycodes LPC, there was a significant interaction between musicianship and congruency [*F*(2,54) = 3.41, *p* = 0.040, ${\text{η}}_{\text{p}}^{2}$ = 0.112]. In musicians with AP, the LPC amplitude was significantly more positive for incongruent than congruent stimuli (*p* < 0.01) while no such differences in ERP amplitude were observed in musicians with RP or in the NM group. Overall, the ERP amplitude was smaller for congruent than incongruent stimuli, [*F*(1,54) = 5.74, *p* = 0.022, ${\text{η}}_{\text{p}}^{2}$ = 0.094]. The main effect of musicianship was also significant [*F*(2,54) = 5.44, *p* < 0.01, ${\text{η}}_{\text{p}}^{2}$ = 0.168], with more positivity of ERP amplitude in NMs than musicians with AP (*p* = 0.012) or RP (*p* = 0.024). There was no significant difference in ERP amplitude between musicians with AP or RP.

## Discussion

In the present study, we used three different auditory versions of the Stroop task to assess the strength of association between the perceived pitch-classes and verbal labels in musicians with RP and AP. We hypothesized that musically trained groups would show greater Stroop interference effects proportionately when presented with incongruent musical notations than non-musicians. We also anticipated that musicians may perform better in absolute terms than non-musicians given the mounting evidence suggesting that musical training enhances executive functions and attentional control (Moreno et al., 2014).

### Behavioral Data: Response Times and Error Rates

Overall, participants were slower and made more errors for incongruent than congruent stimuli. This congruency effect was modulated by lexicon, being stronger for word than for solfège or Germanic keycodes stimuli. Overall, musicians with RP and AP were faster and more accurate than non-musicians. This pattern of results is consistent with many studies showing that musicians often outperform non-musicians in a wide range of auditory tasks involving executive functions and attentional control (Moussard et al., 2016; Alain et al., 2018). However, the analyses of RT data show small or even non-existent congruency effects in musicians for solfège and keycodes versions of the Stroop task. This finding was unexpected and appears inconsistent with the hypothesis that musical training and AP are associated with a strong association between a perceived pitch and a verbal label. One possible explanation for these small and no congruency effects in the solfège and keycodes tasks would be that the musicians were successful in suppressing task-irrelevant information. The pattern of RTs during the word Stroop task is consistent with this possibility, with AP showing the smallest congruency effect, even in terms of accuracy as will be discussed. Further, the reverse Stroop effect findings for keycodes in NMs may occur due to a lack of long-term learning, which makes affirmative association processing or evaluation of unfamiliar label to pitch mappings slower and less accurate than simply ignoring the unfamiliar verbal information and responding to pitch directly in the incongruent keycodes condition.

The analyses of error rate are also consistent with a flexibility of task-relevant attentional control account, with greater accuracy on incongruent trials across the tasks among musicians with RP and AP than non-musicians. Indeed, musicians with AP had virtually no errors in all three versions of the Stroop task, suggesting that they were able to successfully suppress task-irrelevant information and/or competing responses. While musician groups were not immune to incongruence, the findings seem consistent with several studies showing superior performance in musicians compared to non-musicians in a wide range of tasks involving auditory discrimination, executive functions, and attentional control (e.g., Kaiser and Lutzenberger, 2003; Marques et al., 2007; Putkinen et al., 2012; Alain et al., 2018). At the same time, it should be noted that interference effects for words were observed in AP RTs rather than error rate. Interestingly, neutral word conditions were responded to more rapidly and accurately than incongruent stimuli but less rapidly then congruent stimuli by NMs only, while musician RTs did not differ between congruent and neutral English words. This may imply a RT facilitation effect in NMs for congruent trials that has reached fluency in AP and RP, allowing the musicians to name neutral trials with the same fluency NMs seem to name learned percepts such as colors in the color-word Stroop task (Stroop, 1935).

### Neural Correlates: ERP Responses to Congruent and Incongruent Stimuli

The processing of congruent and incongruent stimuli was associated with three temporally and spatially distinct neural signatures. The first modulation peak of interest was directionally positive, occurred at about 200 ms after sound onset, and was larger over midline central and fronto-central areas. In a prior spoken words version of the “low/high” auditory Stroop task (Donohue et al., 2012), a similar neural response was observed around 200 ms. This neural response was found to be significantly attenuated for incongruent compared to congruent sung words but not musical annotations, suggesting that conflicting information in musical domain maybe less salient than in standard language. In the present study, our behavioral data showed reduced congruency effects in the solfège and keycodes tasks. At the same time, the amplitude of the early modulation at 200 ms, or P200, remained comparably unaffected by congruence across both of these tasks. This appears inconsistent with current models positing a strong conditional association between musical notation and pitch, unlike language, which is consistent with the behavioral findings. If both AP and RP musicians were processing musical notation at the same processing strength as words, then one would expect to observe larger and regular congruency effects at that latency for both groups across all tasks as was observed for words across all groups. However, despite a lack of distinct congruency effects on P200 in the music tasks and lack of statistical significance in group main effects, the overall amplitude was nevertheless slightly larger in musicians across tasks and conditions, which seems consistent with studies showing that this modulation may be an important physiological correlate for longer timescale auditory learning (Reinke et al., 2003; Ross and Tremblay, 2009; Alain et al., 2015). The somewhat greater P200 positivity in the RP and AP groups may also imply an advantage in early processing, which might allow the stimulus conflict to be more easily responded to. Certainly, the idea that musical mappings do not produce strong response conflict but musicianship may still allow for greater efficiency in stimulus conflict detection seems to be consistent with both the behavioral and merely observed P200 results. Perhaps, early sensory stream information processing of direction of change or frequency detection from prolonged familiarity to pitch naming causes P200 to be minutely greater in musicians such that the statistically significant congruence effects on P200 are measurably greater in musicians for at least words, which do indeed show evidence of strong associational bonding to pitch information.

The modulation peaking at about 200 ms was followed by another one that peaked at about 450 ms after sound onset, which was characterized by an increased negativity elicited by the incongruent relative to congruent stimuli. This modulation, referred to as the N450, had a broader and more sustained time course than the early P200, and peaked over the left central and fronto-central scalp area. This long lasting negativity could reflect both congruence detection as well as conflict resolution as it remained until a response was made (see also West and Alain, 1999). The N450 amplitude varied as a function of task, being greater for the English word Stroop task than the solfège or keycodes Stroop tasks. This may be due to greater cognitive load related to the semantic effects of the standard language Stroop tasks and greater flexibility between verbal code and musical pitch in terms of associative processing, particularly in the more common RP ability. The Stroop interference effect also decreased when participants were presented with less familiar material such as in the solfège and keycodes tasks. That is, Romanic solemnization or Germanic key lexicons may not possess as strong stimulus to response mappings as the English language (standard auditory Stroop), even for AP. In other words, there is less interference of response in general for pitch naming with musical lexicons than there is for the English language lexicon. Thus, it may also be easier to suppress abstract musical lexicons than concrete referential pitch labels such as low or high. The latter is likely to have a stronger stimulus to response mapping than more abstract musical notations despite prolonged exposure to musical lexicons. However, it may be possible that the significant differences in N450 and also LPC from congruency effects in the keycodes task suggest that only AP seem to experience stimulus conflict though not necessarily response conflict as measured with RTs and accuracy. At the same time, the N450 for both musician groups was significantly lower in amplitude than the NMs, especially in the auditory word Stroop task. This finding may reflect modified thresholds for information conflict detection in the auditory domain for musically trained populations.

The LPC is thought to index conceptual level of representation (West and Alain, 1999). In the present study, its amplitude was modulated by task and musical training, with greater difference between congruent and incongruent stimuli during the auditory word Stroop than musical annotations. Given that the LPC often occurred at the time of or even after a button-press response was made, we reasoned that the conceptual level of representation is a post-response or decision evaluation process, described in previous studies as a semantic re-activation of the lingual item preceding a response (Ergen et al., 2014). The effects of task on this modulation provide some support for such an interpretation because the conceptual level of representation differed between the three tasks, with a stronger representation for words than solfège or keycodes. Interestingly, the modulation was observed in the keycodes task for AP musicians with differences between congruent and incongruent stimuli, while no such modulation was seen in NMs or musicians with RP. This provides converging evidence supporting the notion that AP might yield a conceptual level post-response process for alpha-numeric encodings, which in turn may be modulated by incongruence when there is conflict in information. AP possessors were also significantly different in LPC amplitudes for English words, which may mean they process word forms and alpha-numeric musical encodings similarly but with less response conflict for the musical codes, contrary to our hypotheses. The interaction between musicianship, task, and congruency for the LPC also seems to imply less processing power requirements for the musical tasks, which may show a transfer effect to non-musical tasks in musicians, though both the exogenous and LPC modulations may demonstrate differential use of cognitive resources and possibly behavioral strategy for each group.

## Conclusion

Music can be viewed as another form of language or complex communication medium. Those proficient in music (i.e., musicians) likely develop a lexicon of musical annotations that may share similarities with speech representation. That is, a familiar pitch and musical annotation may automatically trigger a verbal label with a specific meaning. If this is the case, then musicians should experience difficulty in processing incongruent musical stimuli. The present study shows that musicians, even those with AP, exhibited little interference effect at the behavioral level in the two musical versions of the Stroop task. However, the analyses of ERP data show a significant difference between congruent and incongruent in the Germanic keycodes for AP, unlike in the RP or NM groups for this task. This finding suggests that AP possessors, at least at the neural level, might process phonemic equivalents of alpha-numeric encodings of pitch information with a similar associative processing as word forms. Yet, while this processing appears to have semantic properties it is not as powerful as that of standard language. Thus, keycodes appear to have unique processing in music for their mappings to pitch classes in AP. We hypothesize that greater executive function and attentional control associated with playing a musical instrument may help musicians process conflicting information. For this reason, the detection of stimulus conflict may generate attenuated response conflict in musicians compared to NMs, which would help to explain certain discrepancies between the neural and behavioral data in particular parts of our study, along with the more rapid and accurate musician responses. Future research is needed to better understand the apparent contradiction between behavioral and electrophysiological measures in AP. At the same time, the lack of P200 congruency effects in the musical tasks should also be examined by increasing task difficulty and attentional load. Finally, cross-modal transfer of musical training on semantic information may be studied by applying conceptual level processing to domains outside of pitch perception, such as spatialization.

## Data Availability

The datasets generated for this study are available on request to the corresponding author.

## Ethics Statement

The studies involving human participants were reviewed and approved by the Human Research and Ethics Committee at the Baycrest Centre, Toronto, ON, Canada. The participants provided their written informed consent to participate in this study.

## Author Contributions

VS, MT, FR, and CA designed the experiments and interpreted the results of experiments. VS performed the experiments and collected the data. CA and VS analyzed the data. VS and CA drafted the manuscript. All authors edited, revised, and approved the final version of the manuscript.

## Conflict of Interest Statement

The authors declare that the research was conducted in the absence of any commercial or financial relationships that could be construed as a potential conflict of interest.
